# Complete Genome Sequence of the Barley Pathogen *Xanthomonas translucens* pv. translucens DSM 18974^T^ (ATCC 19319^T^)

**DOI:** 10.1128/genomeA.01334-16

**Published:** 2016-12-01

**Authors:** Sebastian Jaenicke, Boyke Bunk, Daniel Wibberg, Cathrin Spröer, Lena Hersemann, Jochen Blom, Anika Winkler, Sarah Schatschneider, Stefan P. Albaum, Roland Kölliker, Alexander Goesmann, Alfred Pühler, Jörg Overmann, Frank-Jörg Vorhölter

**Affiliations:** a Justus-Liebig-University Giessen, Bioinformatics and Systems Biology, Giessen, Germany; b Leibniz Institute DSMZ, German Collection of Microorganisms and Cell Cultures, Braunschweig, Germany; c Center for Biotechnology CeBiTec, Bielefeld University, Bielefeld, Germany; d Agroscope, Molecular Ecology, Zurich, Switzerland

## Abstract

We report here the complete 4.7-Mb genome sequence of *Xanthomonas translucens* pv. translucens DSM 18974^T^, which causes black chaff disease on barley (*Hordeum vulgare*). Genome data of this *X. translucens* type strain will improve our understanding of this bacterial species.

## GENOME ANNOUNCEMENT

The Gram-negative gammaproteobacterium *Xanthomonas translucens* pv. translucens is the causal agent of a bacterial wilt on *Hordeum vulgare* (barley) (1) that is often called black chaff. The strain DSM 18974^T^ (ATCC 19319^T^) was isolated from its host plant in Minnesota, USA. It is the type strain of the species *X. translucens* (2). *X. translucens* strains occur worldwide and infect a broad range of cereals and forage grasses. At the same time, *Xanthomonas* strains are applied in biotechnology to synthesize the exopolysaccharide xanthan (3), a commercial thickening agent. Despite gradually rising numbers of publications, our knowledge about *X. translucens* genomes is still fragmentary. Hence, detailed genome data of *X. translucens* pv. translucens are suitable to advance functional research and deepen our phylogenetic understanding of *X. translucens* and related xanthomonads.

Initially, genomic DNA of *X. translucens* pv. translucens DSM 18974^T^ was extracted (4) to generate a paired-end library that was shotgun-sequenced with a Genome Sequencer FLX (GS FLX) system by means of the 454 Titanium technology (Roche) as described earlier (5). However, the assembly of the resulting data produced a large number of contigs, possibly pointing to the presence of repetitive IS elements. To address this deficiency, a combination of single-molecule real-time (SMRT) and Illumina sequencing technologies was applied. Genomic DNA was extracted applying Qiagen Genomic-tips 100/G according to the manufacturer’s instructions and sequenced on a PacBio RSII instrument (Pacific Biosciences) using P5 chemistry. Genome assembly was performed with the “RS_HGAP_Assembly.3” protocol included in SMRT Portal version 2.3.0, utilizing 49,791 post-filtered reads with an average read length of 12,646 bp. One complete chromosomal contig was obtained and trimmed, circularized, and adjusted to the beginning of the *dnaA* gene. To improve the sequence quality, additional genome data were obtained by sequencing a paired-end TruSeq PCR-free DNA fragment library with an average size of 719 ± 226 bp on a MiSeq instrument (Illumina) in a 2 × 300-bp run. This provided a total of 1,021,089,076 bp in 4,490,068 reads, among them 2,129,085 paired-ends reads. The Illumina reads were mapped with the Burrows–Wheeler aligner (6) onto the contig to obtain the consensus sequence of a circular chromosome of 4,715,357 bp with an average coverage of 223× and a G+C content of 67.7%. Automated genome annotation carried out by means of the GenDB platform (7) with the Prodigal gene prediction software (8) revealed 3,736 protein-coding genes, 54 tRNAs, and two rRNA operons.

Comparative analysis employing the EDGAR software (9) facilitated the identification of a CRISPR array in addition to virulence factor genes, which encoded protein-secretion systems like a *hrp* type III secretion system with transcription activator-like effectors (10) and the polysaccharide xanthan.

### Accession number(s).

This whole-genome shotgun project has been deposited in DDBJ/ENA/GenBank under the accession number LT604072. The version described in this paper is the first version, LT604072.1.
